# Phytochemical profiles and classification of Agave syrups using ^1^H‐NMR and chemometrics

**DOI:** 10.1002/fsn3.755

**Published:** 2018-11-19

**Authors:** Irving O. Velázquez Ríos, Gerardo González‐García, Erika Mellado‐Mojica, Rafael A. Veloz García, Jorge G. Dzul Cauich, Mercedes G. López, María I. García‐Vieyra

**Affiliations:** ^1^ Departamento de Ingeniería Agroindustrial División de Ciencias de la Salud e Ingenierías Universidad de Guanajuato Guanajuato México; ^2^ Departamento de Química División de Ciencias Naturales y Exactas Universidad de Guanajuato Guanajuato México; ^3^ Departamento de Biotecnología y Bioquímica Centro de Investigación y Estudios de Avanzados del IPN Unidad Irapuato Guanajuato México

**Keywords:** agave syrup, antioxidant activity, chemometrics, ^1^H‐NMR, phytochemical screening, total phenolic

## Abstract

**Background:**

Agave syrups are natural sweeteners that are highly desirable for human consumption because they have low glycemic index. In this work, we explored the potential of ^1^H‐NMR‐Chemometrics as a useful tool in the identification and differentiation of Agave syrups. Also, we evaluated the phytochemical screening and antioxidant capacity of Agave syrup compared to other natural sweeteners.

**Results:**

The phytochemical screening stands out for Agave syrups containing higher levels of metabolites with antioxidant activity, mainly saponins, glycosides, and terpenoids. Agave syrup antioxidant activity was in a range from 10% to 53%, while the total phenolic content was from 24 to 300 EAG/100 g, and condensed tannins were between 240 and 1,900 mg CE/g. Additionally, ^1^H‐NMR spectroscopy was used to characterize syrup profiles and chemometrics. PCA group analyses allowed the sweeteners’ classification by origin and kind of Agave.

**Conclusion:**

Thus, we conclude that ^1^H‐NMR and chemometrics can be used for identifying, differentiating, and classifying Agave syrups. Besides, Agave syrups contain significant amounts of antioxidative components and can be considered as an effective source of antioxidant.

1

**Highlights**

- ^1^H‐NMR‐chemometrics is an excellent tool to identify and classify food products.
- Agave syrup can be a promising source of natural phytochemical agents due the presence of metabolites with antioxidant activity
- Agave syrups are natural sweeteners with potential antioxidant capacity.

## 2 INTRODUCTION

Agave syrups are natural sweet substances produced when Agave pines are subjected to a fructan hydrolitic process in order to unfold polysaccharides called agavins (NMX‐FF‐110‐SCFI, 2008) Agavins are fructose polymers where the number of molecules plays an inverse relationship with sweetness: the lower the sweetness, the longer the degree of polymerization.

To produce Agave syrups, plants must be at least 6 years old corresponding to their proximate maturity, as well as their maximum carbohydrates content (Mellado‐Mojica & López, 2013). In the Agave syrups production, the principal agronomic species used are *Agave tequilana* Weber Blue variety and *Agave salmiana* displaying differences in carbohydrate content and composition (Bautista, García, Barboza, & Parra, 2001; Mellado‐Mojica & López, 2013, 2015). Mexican standards do not allow the use of any food additive, ingredient, or sugars from sources other than Agave plants in commercial Agave syrups manufacture (NMX‐FF‐110‐SCFI, 2008).

Agave syrups exhibit high carbohydrate content, mainly composed by fructose (≥60% of the total soluble solids), followed by glucose and with traces of sucrose (Mellado‐Mojica & López, 2015). This carbohydrate composition gives Agave syrups a low glycemic index and makes it sweeter than other syrups containing appreciable levels of glucose and/or sucrose, such as corn and sugarcane (Willems & Low, 2012). Besides fructose and glucose, some fructooligosaccharides (FOS) are also present in some Agave syrups in very smaller amounts as result of incomplete agavin hydrolysis (Mellado‐Mojica, Seeram, & López, 2016).

Nowadays, carbohydrate fingerprinting is a very useful molecular marker of authenticity, adulterants detection, quality, and origin of natural sweeteners; therefore, the determination of glucose, fructose, and sucrose contents, and the oligosaccharides profiles became a method for the determination of quality in honey and syrups (Bueno et al., 2015; Peshev & Van den Ende, 2014; Rizelio et al., 2012).

Due to their relative newness in the market, it is of great importance to study the chemical and phytochemical composition of Agave syrups in order to establish their phytochemical composition and metabolite richness, as well as the development of new strategies for the differentiation of Agave syrups from other natural sweeteners. In addition, the knowledge on their phytochemical composition might be of great importance to human health issues.

In natural sweeteners, phenolic compounds, flavonoids, and carotenoids are reported to be responsible for the antioxidant activity in honeybee and maple syrup (Phillips, Carlsen, & Blomhoff, 2009). In Agave syrups, there is only one report on their antioxidant activity; however, the above investigation was limited to four Agave syrups samples, where three of them were artisanal samples (Olvera, Cardador, & Martín, 2014). Therefore, exploring the potential of commercial Agave syrups as new antioxidant food supplements still needs to be undertaken. Saponins, terpenoids, flavonoids, and tannins have been identified in *Agave sisalana, Agave impressa, Agave ortnithobroma, A. tequilana, Agave angustifolia*, and *Agave americana* species (Ahumada et al., 2013; Dias, Sales, Weingart, & Zarur, 2013; Hamissa et al., 2012) and all or some of these phytochemicals might also be present in Agave syrups.

Nuclear magnetic resonance (NMR) spectroscopy has been widely applied to identify compounds in a wide diversity of food samples such fruit juices, wines, and honeys because it is nondestructive, selective, and capable of detecting a great number components in complex mixtures (Consonni, Cagliani, & Cogliati, 2012; Kosir & Kidric, 2001; Vlahov, Chepkwony, & Ndalut, 2002). Italian honeys were successfully classified based on their NMR spectra and multivariate statistical analysis (Beretta, Caneva, Regazzoni, Bakhtyari, & Facino, 2008; Lolli, Bertelli, Plessi, Sabatini, & Restani, 2008; Schievano, Peggion, & Mammi, 2010). It was suggested that the sugar profile could be used to characterize particular sweeteners used in some samples. NMR spectroscopy coupled to principal component analysis (PCA) can therefore be applied to construct an “identity card” of saccharides for each floral source (Belton et al., 1998; Beretta, Granata, Ferrero, Orioli, & Facino, 2005; Bertelli et al., 2010). The relevance of PCA is mainly to reduce the original data sets to a smaller number of independent variables. PCA can identify trends or characteristics within the NMR data (Beretta et al., 2005; Bertelli et al., 2010).

The aim of this work was to establish the phytochemical screening, total phenolic, and tannin contents, as well as the antioxidant activity of Agave syrups. In addition, ^1^H‐NMR‐PCA was used to differentiate Agave syrups among other natural sweeteners.

## 3 EXPERIMENTAL

### 3.1 Natural sweeteners

A total of 46 natural sweeteners were randomly obtained from different commercial supermarkets and conventional stores from Arandas, Jalisco; Celaya, Guanajuato; Ciudad de México; Guadalajara Jalisco; Irapuato, Guanajuato; Morelia, Michoacán; Oaxaca, Oaxaca; Tuxtla Gutiérrez, Chiapas; Veracruz, Veracruz; and Zacatecas, Zacatecas. In total, 29 Agave syrup samples (A1–A29), 12 honey samples (HB1–HB12), three sugarcane syrup samples (SC1–SC3), and two samples of corn syrup (CS1–CS2) were analyzed and compared. Agave syrup samples include two different Agave species, both from different geographical regions and carbohydrate compositions (Mellado‐Mojica & López, 2015) as described below: 26 syrups from *A. tequilana* (ATS1–ATS26) and three samples from *A. salmiana* (ASS1–ASS3). The samples were stored at room temperature in darkness until analysis.

### 3.2 Syrup extracts

Extracts from each natural sweetener were prepared for phytochemical screening analysis, antioxidant activity, total phenol contents, and condensed tannins (protoantocyanidins) determination. Syrup extracts were obtained according to the protocol described by Chaikham and Prangthip (2014). Briefly, 200 mg of each syrup were placed in a 1.5‐ml Eppendorf tube along with 2 ml of distilled water and 9 ml of absolute ethanol. Samples were mixed for 5 min and then centrifuged at 22,000*g*, for 10 min. Supernatants were separated and collected in a new sample reservoir. Extracts were stored in darkness until their analyses.

### 3.3 Syrups’ phytochemical screening

For the phytochemical screening, the following compounds were tested: saponins, flavonoids, quinones, glucosides, cardiac glycosides, terpenoids, and coumarins following the methodology used by Devika with modifications (Devika & Koilpillai, 2012). Each test was performed in triplicate.

#### 3.3.1 Test for saponins

To 2 ml of syrup extract, 2 ml of distilled water was added and shaken in a graduated cylinder for 15 min length wise. Formation of a 1‐cm layer of foam indicates the presence of saponins.

#### 3.3.2 Test for flavonoids

To 2 ml of syrup extract, 1 ml of 2 N sodium hydroxide was added. Presence of yellow color indicates the presence of flavonoids.

#### 3.3.3 Test for quinones

To 1 ml of extract, 1 ml of concentrated sulfuric acid was added. Formation of red color indicates the presence of quinones.

#### 3.3.4 Test for glycosides

To 2 ml of extract, 3 ml of choloroform and 10% ammonia solution were added. Formation of pink color indicates the presence of glycosides.

#### 3.3.5 Test for cardiac glycosides

To 0.5 ml of extract, 2 ml of glacial acetic acid and few drops of 5% ferric chloride were added. This was under layered with 1 ml of concentrated sulfuric acid. Formation of a brown ring at the interface indicates the presence of cardiac glycosides.

#### 3.3.6 Test for terpenoids

To 0.5 ml of extract, 2 ml of chloroform were added and concentrated sulfuric acid was added carefully. Formation of red brown color at the interface indicates the presence of terpenoids.

#### 3.3.7 Test for coumarins

To 1 ml of extract, 1 ml of 10% NaOH was added. Formation of yellow color indicates the presence of coumarins.

### 3.4 Syrups’ color

The syrups’ color designation was determined according to the United States Standards for Grades of Extracted Honey approved color Pfund scale (USDA, 1985). The sweeteners’ samples were diluted in water in 50% p/v ratio, then heated in a water bath at 50°C. Subsequently, they were centrifuged at 22,000*g* for 3 min to precipitate particles. Absorbance was measured on a BIORAD Mark 10360 microplate reader at λ = 635 nm. The results were expressed according to the Pfund scale (water white, white extra, white, extra light amber, light amber, amber, and dark amber) according to the following formula:

MmPfund=−38.70+371.39∗Absorbance

### 3.5 Antioxidant activity and IC50 from natural sweeteners

Antioxidant activity of the natural sweetener was determined according to the α‐diphenyl‐β‐picrylhydrazyl (DPPH) free radical scavenging method. The DPPH reagent (2,2‐diphenyl‐1‐picrylhydrazyl) was prepared at 150 μM concentration. One hundred and fifty miroliters of each extract were added to 150 μl of DPPH solution placed in a 96‐well microplate. Absolute ethanol was used as a blank. It was subsequently incubated at room temperature in the absence of light and the absorbance was measured on a BIORAD Mark 10360 microplate reader at λ = 517 nm. Readings were taken at 0, 30, and 60 min (Canadanovic et al., 2014).

The antioxidant activity was expressed as:

%InhibitionoftheDPPHradical=(A0−A1(30min))/A0∗100

The IC50 is the inhibitory activity expressed by the amount of sample per milliliter (g/ml) of solution that achieves 50% inhibition of DPPH free radicals. Three different concentrations 0.1, 0.2, and 0.5 g/ml of syrup from ethanol–water solution 82% and 18%, respectively, of each extract were prepared; 0.1 ml of each solution was mixed with 3.9 ml of 0.1 mM DPPH. Immediately, zero time and after 30 min reading were made, at λ = 515 nm. A linear regression was applied to the graph of concentration of the extract versus % inhibition of the DPPH radical. Low IC50 values represent strong antioxidant activities (García, Aguilar, Soto, Nieto, & Kite, 2012; Ruiz, Venegas, Díaz, & Rodríguez, 2012).

### 3.6 Total phenol contents in sweeteners

The total phenol content was determined according to the methodology reported by Pelvan, Alasalvar, and Uzman (2012) with some modifications. The syrup extracts were prepared weighing 0.1 g of sample in 1 ml of distilled water and centrifuged at 22,596*g* for 3 min. Thirty microliters of the extract was mixed with 150 μl of the Folin–Ciocalteu reagent for 5 min; then 120 μl of calcium carbonate was added and incubated at room temperature for 2 hr. Absorbance was measured on a microplate reader (iMarkTM BIO‐RAD) at λ = 750 nm. Gallic acid (GA) was used as the standard to perform the calibration curve of 0 to 200 mg/L concentrations. Total phenols were expressed as milligrams of gallic acid equivalents per gram of extract (mg of GAE/g of extract).

### 3.7 Condensed tannins in natural sweeteners

The condensed tannin contents were estimated according to a modified protocol employed by Tili et al. (2015). Ten microliters of ethanolic extract were mixed with 197 μl of 4% ethanol–vanillin solution and 99 μl of concentrated sulfuric acid incubated at room temperature and subsequently the absorbance was measured at λ = 490 nm. Condensed tannins were expressed in milligrams of catechin equivalents per gram of sample (EC)/g.

### 3.8 ^1^H‐NMR spectroscopy‐PCA from natural sweeteners

Nuclear magnetic resonance spectra of syrup samples were acquired with a Varian/Agilent 600 MHz AR Premium COMPACTTM spectrophotometer; all NMR experiments were performed at 300 K. The ^1^H‐NMR spectra were measured at 300 K and 599.77 MHz frequency using D_2_O as solvent and as an internal reference; the residual HOD signal was used at 4.9 ppm. The pulse of π/2 employed was 8.7 μs, relaxation time of 15 s with 16 repetitions.

The principal component analyses of the spectra of ^1^H‐NMR were obtained by analyzing the carbohydrate region in the MestreNova 10.0.2 software, measuring the area of the emitted signals in the peaks of the carbohydrate region. Twenty‐six samples of Agave syrups (*A. tequilana*), three of Agave syrups (*A. salmiana*), 12 of honey, three of sugar cane syrup, and two of corn syrup were analyzed. Once the data were collected, the main components were analyzed in the statistical software STATGRAPHICS Plus 5.1, analyzing sweetener and later all the data together.

### 3.9 Statistical data analysis

Analysis of variance was realized in the statistical software Infostat, with a confidence level of 95%. The means comparison was made with the Mean Significant Difference (LSD Fisher) method with *α* = 0.05, with a confidence level of 95% (*p* < 0.05). Each test was performed in triplicate.

## 4 RESULTS AND DISCUSSION

### 4.1 Phytochemical screening

Phytochemical compounds such as saponins, flavonoids, quinones, glycosides, cardiac glycosides, terpenoids, and coumarins are known as nutraceutical compounds due to their medicinal importance.

The phytochemical screening from the ethanolic extracts of naturals sweeteners was established according to a colorimetric qualitative scale where the presence and abundance (appreciable amounts [++], moderate amounts [+], and the absence [−]) of the natural products are indicated. Saponins, flavonoids, quinones, glucosides, cardiac glycosides, terpenoids, and coumarins were determined in all the ethanolic extracts of natural sweeteners (Table 1).

**Table 1:** Compositional profile of natural sweeteners

NS	Saponins	Flavonoids	Quinones	Glucosydes	Cardiac Glucosydes	Terpenoids	Coumarins
ATS 1	++	+	+	+	++	+	+
ATS2–ATS3	+	−	+	+	++	−	+
ATS9–ATS10	++	+	+	+	+	−	+
ATS13	++	+	+	+	++	−	−
ATS14	+	−	−	+	+	−	−
ATS 15–26	+	−	+	+	++	−	+
ASS1–ASS3	++	+	+	+	++	++	+
HB1	+	+	+	+	+	+	+
HB3	+	+	+	+	+	−	+
HB6	+	+	+	+	+	+	+
HB4–5; HB9–12	+	−	+	+	+	++	+
SC1–SC3	+	+	+	+	+	+	+
CS1–CS2	−	+	−	+	++	−	−

*Note*

NS: natural sweetener; ATS: *Agave tequilana* syrup; ASS: *Agave salmiana* syrup; HB: honey; SC: sugar cane syrup; CS: corn syrup; (−): absent; (+): moderately present; (++): appreciable amount.

The agave syrups exhibited appreciable amounts of saponins and cardiac glucosides, and terpenoids followed by glucosides, quinones, flavonoids, and coumarins in moderate amounts. In the case of *A. tequilana* syrup (ATS), flavonoids and terpenoids were only detected in few samples. On the other hand, *A. salmiana* syrup (ASS) displayed positive colorimetric reaction for all the evaluated compounds; this could indicate highest richness of phytochemical compounds. The extracts ATS11 and ATS12 were not made due to lack of sample. The extracts with same phytochemical profile were grouped (Table 1).

The phytochemical screening from sugarcane syrup and honey samples showed the presence of all mentioned compounds in moderate amounts, whilst corn syrup presented the lowest phytochemical composition of all.

The above findings showed the potential of Agave syrups as a new phytochemical and natural source of these valuable compounds.

The presence of natural products in the four different sweetener groups was identified, and in some cases, higher presence of these natural products was observed than in others. In general, the samples presented positive results for each of the seven compound groups analyzed in this work, except for two samples of ATS (ATS1 and ATS2) and two samples of honey (HB4 and HB5) that gave negative results for flavonoids and terpenoids (Table 1). In the tests of saponins, cardiac glycosides, and terpenoids, the agave syrups presented greater color intensity in the reaction, which could indicate a higher amount of these compounds with respect to other evaluated sweeteners. However, even within the Agave syrups samples, mainly ATS, there are differences in composition since at least four of them presented very different results (Table 1).

The phytochemical characterization of the *A. salmiana* plants showed in the preliminary tests the presence of natural products such saponins and flavonoids; this is in agreement with the results obtained in this work, but at the same time contrasted the detections of some secondary metabolites like cardiac glycosides and coumarins (Fernández Anderson, 2005; Romero, Osorio, Flores, Robledo, & Mora, 2015). This could be due to the different protocols used to obtain the extracts in this study, since the Agave plants received a thermal treatment to obtain the syrups.

Corn syrups had the lowest presence of natural products, showing activity only for three groups: flavonoids, glycosides, and cardiac glycosides (Table 1). Nevertheless, CS was unique by the high content of cardiac glycosides observed.

In general, the four different sweeteners presented potential as source of natural components. Agave syrups might be highlighted due to the greatest amounts of saponins, cardiac glycosides, and terpenoids with respect to other evaluated sweeteners.

Finally, several natural products have been found in Agave species, for example, the presence of saponins with a content from 1.17 to 100 g in samples of Agave obtained from *Agave atrovirens* during fermentation (Can et al., 2015).

### 4.2 Color

The color in syrups is partly related to their content of phytochemicals with antioxidant activity such carotenoids and flavonoids (Chaikham & Prangthip, 2014). Syrups were diluted to 50% (w/v) with distilled water and their absorbance was determined. The absorbance of the used dilution presented values from 164 to 16 mm/Pfund. *A. salmiana* syrups being the sweeteners with greater color intensity were classified as “Dark amber” and the corn syrups with lowest intensity were classified as “Extra white” (Table 2). The colors in all these natural sweeteners were in accordance with data described by Mellado‐Mojica and López (2013, 2015) where ATS syrups were classified in a range from white to clear amber and ASS syrups exhibited a dark amber color.

**Table 2:** Color range of natural sweeteners

NS	Color (mm/Pfund scale)
ATS1	Light amber (59)
ATS2	White (30)
ATS3	White (27)
ATS4	White (31)
ATS5	White (19)
ATS6	White (24)
ATS7	Light amber (57)
ATS8	White (22)
ATS9	White (27)
ATS10	White (31)
ATS11	Extra light amber (45)
ATS12	White (29)
ATS13	White (29)
ATS14	White (27)
ATS15	White (26)
ATS16	Extra light amber(39)
ATS17	White (19)
ATS18	White (24)
ATS19	White (30)
ATS20	White (31)
ATS21	White (33)
ATS22	White (25)
ATS23	Extra light amber (47)
ATS24	White (27)
ATS25	White (27)
ATS26	White (29)
ASS1	Dark amber (139)
ASS2	Dark amber (117)
ASS3	Dark amber (129)
HB1	White (26)
HB2	Amber
HB3	Amber
HB4	Extra light amber (40)
HB5	Extra light amber (35)
HB6	Dark amber (119)
HB7	Light amber (61)
HB8	Light amber (58)
HB9	Light amber (57)
HB10	Light amber (65)
HB11	Dark amber (124)
HB12	Amber
SC1	Dark amber (143)
SC2	Dark amber (159)
SC3	Dark amber (164)
CS1	Extra white (16)
CS2	Extra white (17)

*Note*

NS: natural sweetener; ATS: *Agave tequilana* syrup; ASS: *Agave salmiana* syrup; HB: honey; SC: sugar cane syrup; CS: corn syrup.

On average, ASS showed higher mm/Pfund (128.33 mm/Pfund) considering that the color of syrup is given partly by antioxidant components; ASS became a good antioxidant candidate.

The physicochemical properties, antioxidant capacities, and phenolic profiles in honeys from Turkey have been reported (Alves, Ramos, Goncalves, Bernardo, & Mendes, 2013). The authors found a relationship between the color and the antioxidant capacity concluding that darker honeys present higher antioxidant activity. Similarly, Alves et al. (2013) described similar findings where dark‐colored Portuguese honeys had higher antioxidant activities when compared to light‐colored honeys.

### 4.3 Antioxidant activity and IC50

The antioxidant activity of natural sweeteners was determined as the percent of free radical scavenging compared to the free radical scavenging of the DPPH reagent. The antioxidant activity of natural sweeteners was in average as described below: ATS 23.56%, ASS syrups 28.33%, HB 24.16%, SC 25.66%, and CS with 8.6%. The relationship between color and antioxidant capacity was also observed in this work (Alves et al., 2013; Meda, Lamien, Romito, Millogo, & Nacoulma, 2005). Darker syrups (*A. salmiana* and sugarcane syrups) had the highest antioxidant activities with an average of 28.33% and 25.66%, respectively, whereas lighter syrups (CS) exhibited the lowest antioxidant capacity with an average of 8.7% (Table 3).

**Table 3:** Antioxidant activity, total phenol content, condensed tannin content, and IC50 of natural sweeteners

NS	AA (%)	TPC (GAE)/100 g)	CTC (mg CE/g)	IC50
ATS1	14.37 ± 1.53	300.09 ± 62.93	240.00 ± 35.36	0.684
ATS2	36.18 ± 0.93	33.30 ± 10.10	691.67 ± 92.38	0.388
ATS3	24.22 ± 2.06	25.94 ± 2.43	600.56 ± 61.58	0.443
ATS4	24.16 ± 1.92	28.20 ± 7.91	519.44 ± 32.03	0.565
ATS5	24.97 ± 2.98	28.77 ± 3.92	408.33 ± 80.14	1.1
ATS6	29.51 ± 10.74	24.36 ± 5.47	998.33 ± 65.66	0.341
ATS7	25.12 ± 10.53	65.18 ± 5.29	1,018.33 ± 51.85	0.601
ATS8	18.21 ± 4.70	49.08 ± 5.18	818.33 ± 23.57	0.751
ATS9	27.30 ± 1.70	35.75 ± 1.15	853.33 ± 11.79	0.452
ATS10	25.67 ± 2.40	31.35 ± 5.87	306.67 ± 7.07	0.432
ATS11	16.86 ± 1.02	50.03 ± 3.51	247.22 ± 94.28	0.872
ATS12	29.25 ± 0.47	49.96 ± 4.90	866.67 ± 2.36	0.334
ATS13	18.21 ± 13.70	22.42 ± 4.61	396.67 ± 32.72	0.726
ATS14	10.46 ± 0.36	31.03 ± 5.00	468.33 ± 37.71	0.407
ATS15	22.33 ± 2.70	28.33 ± 6.51	700.00 ± 209.78	0.258
ATS16	52.2 ± 5.59	29.46 ± 3.05	529.44 ± 15.03	0.199
ATS17	22.75 ± 0.60	42.16 ± 1.36	671.67 ± 4.71	3.166
ATS18	30.59 ± 13.32	51.53 ± 10.94	667.22 ± 67.36	0.54
ATS19	30.28 ± 0.81	30.97 ± 7.86	520.56 ± 47.18	0.841
ATS20	11.20 ± 2.51	70.34 ± 3.49	348.33 ± 26.46	1.161
ATS21	30.19 ± 21.44	57.01 ± 3.4	663.33 ± 1.92	0.716
ATS22	11.77 ± 3.51	44.81 ± 3.18	945.00 ± 3.85	0.425
ATS23	39.64 ± 8.48	28.70 ± 4.1	976.67 ± 25.02	1.238
ATS24	40.57 ± 9.81	39.90 ± 3.16	423.33 ± 48.11	0.3
ATS25	36.38 ± 17.03	32.98 ± 7.35	808.33 ± 44.10	7.34
ATS26	28.43 ± 19.58	46.44 ± 5.28	645.00 ± 65.43	0.724
ASS1	16.12 ± 0.51	116.50 ± 30.23	572.78 ± 82.75	0.377
ASS2	25.38 ± 1.66	230.21 ± 84.86	1,598.33 ± 23.57	0.394
ASS3	34.22 ± 4.21	253.30 ± 84.66	1,183.33 ± 25.93	0.302
HB1	26.38 ± 7.83	23.42 ± 3.00	187.22 ± 67.69	0.67
HB2	24.97 ± 5.37	21.72 ± 2.36	287.22 ± 90.02	0.53
HB3	8.79 ± 4.85	30.96 ± 2.28	1,140.00 ± 7.07	0.522
HB4	24.93 ± 8.98	21.91 ± 1.26	357.22 ± 145.39	0.574
HB5	28.88 ± 7.87	19.90 ± 1.86	1,426.67 ± 82.50	0.41
HB6	26.82 ± 6.27	38.26 ± 1.64	280.00 ± 21.21	0.253
HB7	26.44 ± 1.36	51.28 ± 0.65	342.78 ± 70.74	0.225
HB8	26.18 ± 1.97	23.04 ± 1.96	443.33 ± 11.79	0.345
HB9	43.19 ± 3.12	33.17 ± 6.81	570.00 ± 21.17	0.263
HB10	29.97 ± 5.99	31.41 ± 4.12	603.33 ± 171.28	0.411
HB11	23.33 ± 2.05	107.95 ± 7.93	1,383.33 ± 49.50	0.526
HB12	27.25 ± 2.65	31.28 ± 0.82	365.00 ± 0.00	0.748
SC1	26.87 ± 2.20	185.22 ± 39.3	693.33 ± 11.79	0.251
SC2	15.15 ± 1.78	180.34 ± 7.13	683.89 ± 12.62	0.39
SC3	36.33 ± 4.62	184.74 ± 1.2	660.00 ± 25.93	0.404
CS1	9.15 ± 3.63	11.91 ± 1.42	184.66 ± 89.88	35.80
CS2	8.71 ± 1.35	16.00 ± 1.47	145.56 ± 51.89	30.82

*Note*

NS: natural sweetener; ATS: *Agave tequilana* syrup; ASS: *Agave salmiana* syrup; HB: honey; SC: sugar cane syrup; CS: corn syrup; AA: antioxidant activity; TPC: total phenol content; CTC: condensed tannin content; GAE: gallic acid equivalents; CE: catechin equivalents.

*Agave tequilana* syrups showed great variability with respect to this parameter; the above could be probably due to the botanical origin of the sweeteners and processing, and also due to problems of product standardization, meaning quality. However, ATS as well as ASS may be good antioxidant syrup candidates.

The IC50 in general agreed with the results obtained on the antioxidant activity determination by the percentage of free radical scavenging DPPH since the sample with the highest antioxidant activity, ATS‐16 with 52.20% free radical uptake, was the sweetener needing less sample amount to reduce free radicals DPPH to 50% (0.199 g) according to the results obtained in the IC50. On the other hand, CS‐2 sample had the lowest antioxidant activity (8.71%) and therefore, 30.82 g syrup is required to reduce free radicals DPPH to 50%. This proportion is conserved for most of the samples analyzed (Table 3).

In honey bees, the antioxidant capacity was attributed to the combined activity of a wide range of natural compounds such phenolics, organic acids, Maillard reaction products, and probably other minor components (Daglia, 2012). Thereby, the antioxidant activity of Agave syrups could be due to similar natural compounds, so it would be of great importance to characterize and identify these phytochemical compounds in these new sweeteners.

### 4.4 Total phenols

The natural sweeteners displaying the darkest color also showed the highest total phenolic compound amounts as well as major antioxidant capacity, *A. salmiana* and sugarcane syrups (253.30 and 185.22 EAG/100 g, respectively) (Table 3). These results suggest that phenolic compounds in these natural sweeteners might be responsible for the antioxidant activity observed (Table 3).

In some studies where the antioxidant activity and phenolic content were determined, such as berries, fruit wines, and liqueurs, no relationship was found between the two parameters (Ruiz et al., 2012). This information agrees with our results; we did not find statistically relationship (data no show) between phenolic and antioxidant activity. On the other hand, it is very useful to associate individual phenolic compounds with antioxidant activity because their particular structural characteristics are able to neutralize free radicals more easily (Ruiz et al., 2012).

### 4.5 Determination of condensed tannins

Proanthocyanidins are compounds composed of flavyl units, containing carbohydrates and amino acid residues, and also having different degrees of condensation and called condensed tannins.

The range of content of tannins was 1,598.33 for Agave syrups (ASS 2) and 145.56 for corn syrup (CS2) expressed in mg EC/g of sample; all other syrups were ranked between these values. On average, corn syrups were the sweeteners with the lowest content of proanthocyanidins (145.56 ± 51.89); this result is in accordance with those obtained in previous tests (Table 3).

The results obtained in the quantification of proanthocyanidins presented the same behavior than the antioxidant (Supplementary table). In this case, again corn syrups standout for having the lowest content of proanthocyanidins with an average value of 165.11 mg EC/g with respect to the rest of the sweeteners. Corn syrups had the lowest antioxidant activity and the lowest total phenol content so it is assumed that the antioxidant activity of corn syrups might mainly be due to their content of protoantocyanidins. Through this study, different natural products associated with corn syrup would reveal the main causes of the antioxidant activity of the same. There is no literature report supporting this information on corn syrups.

Among Agave species, ASS exhibited major phytochemical diversity with respect to ATS. *A. salmiana* syrups presented the highest content of proanthocyanidins (1,118.03 mg EC/g on average) when compared to ATS (615.51 mg EC/g), thereby resulting in better antioxidant activity. However, it is important to note that in ATS, proanthocyanidins amounts presented a wide range of values; therefore, ATS also displays a good potential as an antioxidant for human consumption.

### ^3.6^ ^1^H‐NMR spectroscopy‐PCA of natural sweeteners

Mellado‐Mojica and López (2013, 2015) reported the physicochemical properties and carbohydrate profiles of Agave syrups. The authors describe that glucose, fructose, and sucrose amounts were the main differences between syrups from different Agave species. They also mentioned that oligosaccharide type and profile play an important role in the differentiating *A. tequilana* and *A. salmiana* syrups.

**Figure 1:**
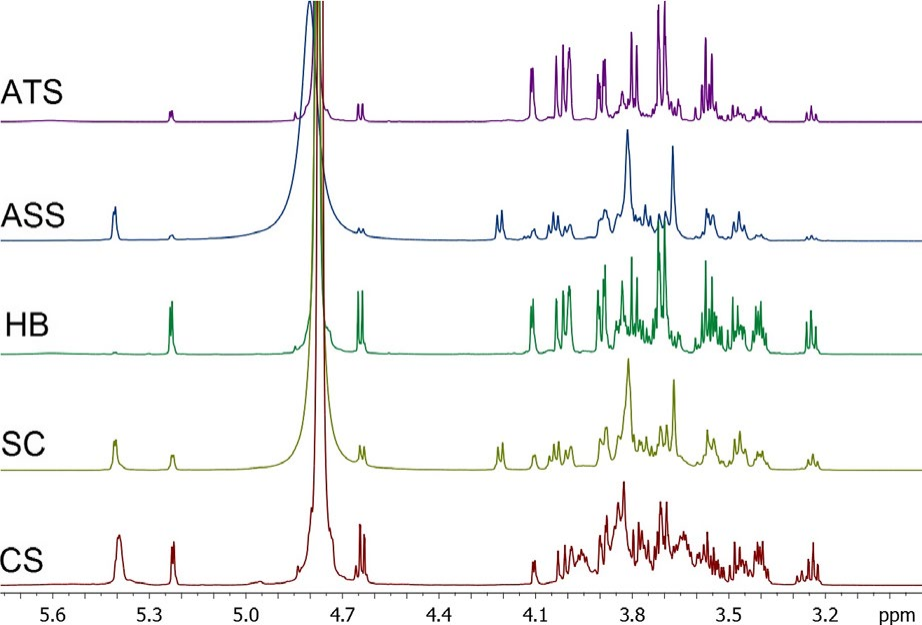
^1^H‐NMR spectra of different sweeteners. ^1^H‐NMR spectra of the carbohydrate region (3.2–5.4 ppm). ATS:* Agave tequilana* syrup; ASS:* Agave salmiana* syrup; HB: honey; SC: sugarcane syrup; CS: corn syrup

In this work, we analyzed the ^1^H‐NMR spectra of the Agave syrups and other natural sweeteners (Figure 1). ^1^H‐NMR spectra reveal differences among natural sweeteners; however, some signals are also common (Figure 1). In general, *A. tequilana* syrups showed greater intensity signal emitted by the peaks, at 4.0 ppm for fructose, and 3.8 and 3.7 ppm that correspond to sucrose. *A. salmiana* syrup signals had the same tendency; however, it stands out for presenting an additional signal at 5.4 ppm, corresponding to sucrose.

Previously, carbohydrate identification of the same five sweeteners types using thin layer chromatography (TLC) shows that *A. tequilana* syrup (ATS) had a high content of fructose and also contained traces of fructooligosaccharides (kestose and nystose); the authors mentioned this formed during the hydrolysis of fructans as a consequence of the thermal process (Mellado‐Mojica & López, 2013, 2015). The corn syrup and sugar cane presented glucose and maltooligosaccharides, and the sugarcane syrup also presented sucrose. The presence of glucose, fructose, maltose, and maltotriose is a characteristic mainly of honey.

The ^1^H‐NMR spectra were similar in all different sweeteners, emphasizing the intensity of signals in different positions of the spectrum; this is due to the particular carbohydrate content in each syrup. It is possible to differentiate a sweetener from another using the ^1^H‐NMR profiles. However, it is indispensable to support NMR data with multivariable methods such component analyses, which allows syrups to be grouped according to their particular characteristics.

**Figure 2:**
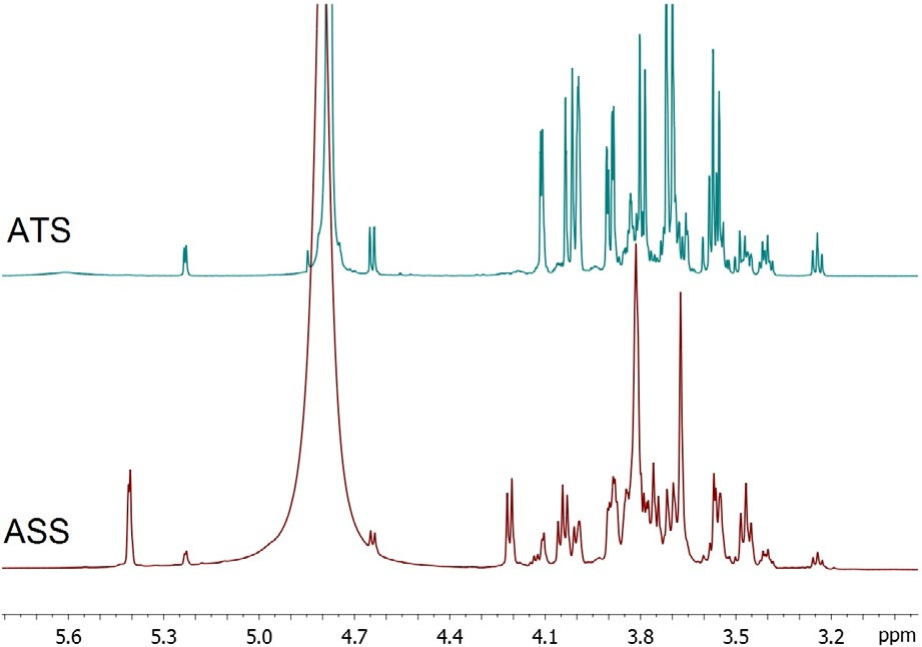
^1^H‐NMR spectra from Agave syrups. ^1^H‐NMR spectra of the carbohydrate region (3.2–5.4 ppm) ATS:* Agave tequilana* syrup; ASS:* Agave salmiana* syrup

Figure 2 shows the ^1^H‐NMR spectra of the Agave syrups where differential peaks between samples in different regions along the spectrum are clearly observed.

**Figure 3:**
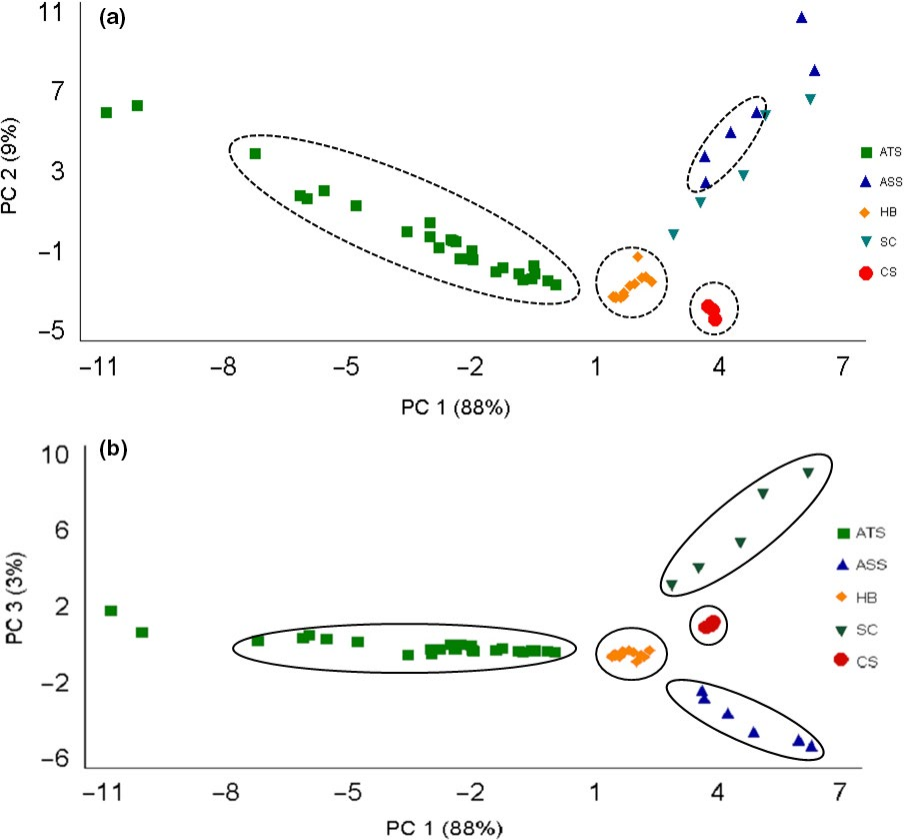
PCA analysis of the ^1^H‐NMR spectra of the carbohydrate region of different sweeteners. ATS:* Agave tequilana* syrup; ASS:* Agave salmiana* syrup; HB: honey; SC: sugarcane syrup; CS: corn syrup; PC 1–3: principal component 1–3

The PCA permitted a small number of linear combinations of the 48 peaks obtained by ^1^H‐NMR from the carbohydrate region that account for the majority of the variability in the data to be obtained. In this case, three principal components were extracted since these components have eigenvalues greater than or equal to 1.0. Together they explained 97% of the variability of the original data (Figure 3).

Principal component analysis allowed the identification and classification of each and every sweetener using only the carbohydrates region of the ^1^H‐NMR. The sweeteners studied in this work were grouped successfully. Agave syrups were separate from other non‐Agave sweeteners. However, *A. tequilana* syrups presented a slight variation between the different samples, due that it was not completely grouped. The same behavior was observed for sugarcane syrup; it is worth mentioning that when plotting PC1 against PC2 (Figure 3a) for sugarcane and *A. salmiana* sweeteners, the plots are very near/overlapping, which means that they share a similar carbohydrate profile, mainly similarities on sucrose content (Mellado‐Mojica & López, 2015). In addition, when darker sweeteners were compared to the other sweeteners, these two syrups were classified as “Dark Amber.”

It is known that sugars are the main component of honey and syrups and the possibility to analyze these components would help establish the qualitative characteristics and authenticity of these products (Consonni, Cagliani, & Cogliati, 2013; Kortesniemi et al., 2016). The PCA 1–3 plot (Figure 3b) shows a clearer grouping of these natural sweeteners depending on their origin, differentiating even among Agave species, demonstrating the potential of ^1^H‐NMR for a good differentiation and classification of foods. The global sugar composition in reference to other natural sweeteners played an important role in the discrimination (Kortesniemi et al., 2016; Lolli et al., 2008).

The ^1^H‐NMR spectra coupled with principal component analyses reveals that there is a difference in carbohydrate profiles between Agave syrups relative to other sweeteners; there are even differences among Agave syrups according to the species.

**Figure 4:**
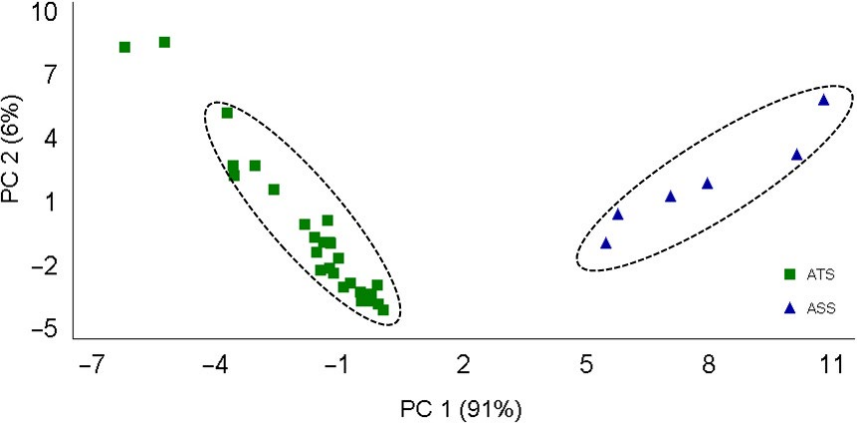
PCA analysis of the ^1^H‐NMR spectra of the carbohydrate region from Agave syrups. ATS:* Agave tequilana* syrup; ASS:* Agave salmiana* syrup. PC 1–2: principal component 1–2

Figure 4 presents a PCA of the Agave syrups (ATS‐ASS) clearly showing a difference among these two species; it was observed that Agave syrups differ in composition and carbohydrate content.

## 5 CONCLUSION

Agave syrups showed a greater phytochemical potential than other sweeteners due to the presence of more natural compounds with antioxidant activity. They were also different in carbohydrate profile with respect to other sweeteners, even in species of Agave. The differences in the chemical composition also occur within the same samples of *A. tequilana* syrups; this difference is due to different times used in the cooking process of the Agave.

Among the Agave syrups, ASS showed the highest phytochemical potential due to their higher antioxidant activity, higher content of total phenols, and proanthocyanidins compared to ATS. In addition, ASS also showed greater homogeneity in the color of the samples.

The color of the sweeteners was related to the content of pigments with antioxidant activity since darker sweeteners had higher antioxidant activity, content of phenols, and proanthocyanidins.

The ^1^H‐NMR spectra of Agave syrups and comparative sweeteners were obtained. ^1^H‐NMR data coupled to multivariate methods such as principal component analysis (PCA) allowed the identification and classification of Agave syrups as well as differentiation with respect to other sweeteners.

## CONFLICT OF INTEREST

The authors declare that they do not have any conflict of interest.

## ETHICAL STATEMENT

This study does not include any animal or human tests.
