# Supplementary material for: Phytochemical profiles and classification of Agave syrups using 1H‐NMR and chemometrics
Source: Food Sci Nutr. 2018 Nov 19;7(1):3–13. doi: 10.1002/fsn3.755 (PMC6341176; doi:10.1002/fsn3.755)
Supplement: Supplementary file 1 [file FSN3-7-3-s001.docx]

**Additional information**

**Tabla 4. Population means comparison**

| **NS** | **Antioxidant**  **Activity** | **Total**  **Phenol** | **Condensed**  **Tannin** |
| --- | --- | --- | --- |
| **ATS** | 21.69 **B** | 48.00 **B** | 674.96B **C** |
| **ASS** | 33.86 **A** | 200.00 **A** | 1118.15 **AB** |
| **HB** | 29.05 **A** | 36.19 **B** | 615.51 **C** |
| **SC** | 37.80 **A** | 185.22 **A** | 679.07 **BC** |
| **CS** | 6.18 **C** | 13.96 **C** | 1651.13 **A** |

*means with a letter in common are not significantly different (P <0.05)
